# A specific protocol of autologous bone marrow concentrate and platelet products versus exercise therapy for symptomatic knee osteoarthritis: a randomized controlled trial with 2 year follow-up

**DOI:** 10.1186/s12967-018-1736-8

**Published:** 2018-12-13

**Authors:** Christopher Centeno, Mitchell Sheinkop, Ehren Dodson, Ian Stemper, Christopher Williams, Matthew Hyzy, Thomas Ichim, Michael Freeman

**Affiliations:** 1 Centeno-Schultz Clinic, 403 Summit Blvd Suite 201, Broomfield, CO 80021 USA; 2 Regenexx, LLC, Des Moines, IA USA; 3 Regenerative Pain Center, Des Plaines, IL USA; 4 Immune Advisors, LLC, San Diego, CA USA; 5 CAPHRI, School of Public Health and Primary Care, Maastricht University, Maastricht, Netherlands

**Keywords:** Knee osteoarthritis, Bone marrow concentrate, Articular cartilage, Regenerative therapy, Exercise therapy

## Abstract

**Background:**

Cell-based therapies have shown promise for the treatment of knee osteoarthritis (OA). The current study compared exercise therapy to autologous bone marrow concentrate (BMC) and platelet products for knee OA treatment.

**Methods:**

Patients with symptomatic knee OA (N = 48) were randomized into either an exercise therapy control group or treatment group with injection of autologous BMC and platelet products. Patients in the control group could crossover to BMC treatment after 3 months. Clinical outcomes were documented at baseline and at 6-weeks, 3, 6, 12 and 24 months, including the Knee Society Score (KSS), Pain Visual Analogue Scale, Short Form-12 Scales (SF-12), and Lower Extremity Activity Scale (LEAS).

**Results:**

All patients in the exercise group crossed over to receive BMC treatment after 3 months (N = 22 crossover). At 3 months, KSS-knee, SF-12 Physical, and LEAS improved significantly in the crossover group compared to exercise, similar to significant improvements on KSS-knee and LEAS for the treatment group (N = 26) compared to exercise group at 3 months. After BMC treatment, patients’ clinical outcome scores (except SF-12 Mental Health), were significantly improved through the 2-year follow-up compared to baseline. No serious adverse events were reported.

**Conclusion:**

The use of image-guided percutaneous BMC with platelet products yielded better results than exercise therapy as an effective alternative therapy for patients with symptomatic moderate to moderate-severe osteoarthritis of the knee.

*Trial registration* NCT02034032. https://clinicaltrials.gov/ct2/show/NCT02034032. Registered 13 January 2014

## Background

Osteoarthritis (OA) is one of the most common causes of chronic joint pain. In the United States, symptomatic OA affects more than 50 million adults, resulting in annual costs due to medical expenses and lost wages exceeding $100 billion [1, 2].

Conservative treatment options for painful knee OA aimed at controlling pain and improving function, are often unsatisfactory. Treatment modalities include pharmacologic agents, physical therapy, and injections. The most common pharmaceutical therapies for knee OA include non-steroidal anti-inflammatory drugs (NSAIDs), which are not curative and associated with side effects when used long term, including upper gastrointestinal complications and increased risk of cardiovascular events [3]. Exercise or physical therapy including aerobic walking and strengthening exercises has been shown to improve function and reduce pain compared to control groups [4, 5], while aquatic therapies provide short term benefits [6]. Minimally invasive injection procedures for OA such as corticosteroid injections only demonstrate modest clinical benefits without altering disease progression and may increase the rate of cartilage loss [7].

The only definitive treatment option for end-stage knee OA is arthroplasty. Post-operative complications include deep vein thrombosis and neuropathy, and up to 34% of patients report persisting moderate-to-severe pain [8, 9]. Hence, cell-based therapy including platelet rich plasma (PRP) and bone marrow concentrate (BMC) [10, 11] have been discussed as less invasive options. Autologous BMC contains mesenchymal stem cells (MSCs), platelets, and other cells with healing and regeneration potential (e.g. hematopoietic stem cells and macrophages) [12, 13]. Multiple studies have demonstrated encouraging results for the use of BMC for OA in human populations, although few controlled trials exist [14, 15].

In the present investigation we describe a randomized controlled trial of a specific protocol of image guided percutaneous injection of a combination of BMC and platelet products versus an exercise therapy regimen among patients with moderate knee osteoarthritis. We hypothesized that a specific protocol of BMC and platelet products would improve clinical outcomes more than exercise therapy alone.

## Materials and methods

### Study design

Study patients were recruited from January 2014 to January 2016 from an outpatient orthopedic practice in Chicago, IL. Eligible patients had knee OA grade II or III according to Kellgren–Lawrence (KL) classification [16] (see Table 1 for inclusion and exclusion details). Patients were informed of the study protocol and randomization to one of two groups in a 1:1 ratio using a computer-generated randomization program with enrollment randomization envelopes blinded until time of enrollment by study coordinator. The BMC treatment group received an injection of autologous BMC and platelet products, and the control group underwent a home exercise therapy program following instruction in knee strengthening and stability exercises. Patients in the exercise group were offered the opportunity to cross over to the treatment group after 3 months of exercise therapy, as a method to aid in study recruitment and retention. Patients were followed for 2 years after receiving BMC treatment. The study protocol underwent review and approval through International Cellular Medicine Society IRB (OHRP Registration #IRB00002637).

**Table 1:** Inclusion and exclusion criteria

Inclusion criteria
Men or women aged 18–70
Diagnosis of knee osteoarthritis
Kellgren–Lawrence (KL) classification of grade II or III OA severity
Exclusion criteria
BMI > 30
Knee flexion < 110º
Knee varus > 12º
Knee valgus > 15º
Instability as demonstrated by > 2 mm translation upon physical examination
Knee flexion contracture greater than 15º
History of ACL reconstruction or evidence of complete or partial ACL disruption
Knee Society Score < 65
History of septic arthritis within the last 5 years
History of knee surgery within the last 6 months
Currently experiencing low back pain with radiculopathy
History of immunosuppressive disease or chemotherapy in last 5 years
History of systemic neurological disease
Positive HIV serology or chronic hepatitis

**Fig. 1:**
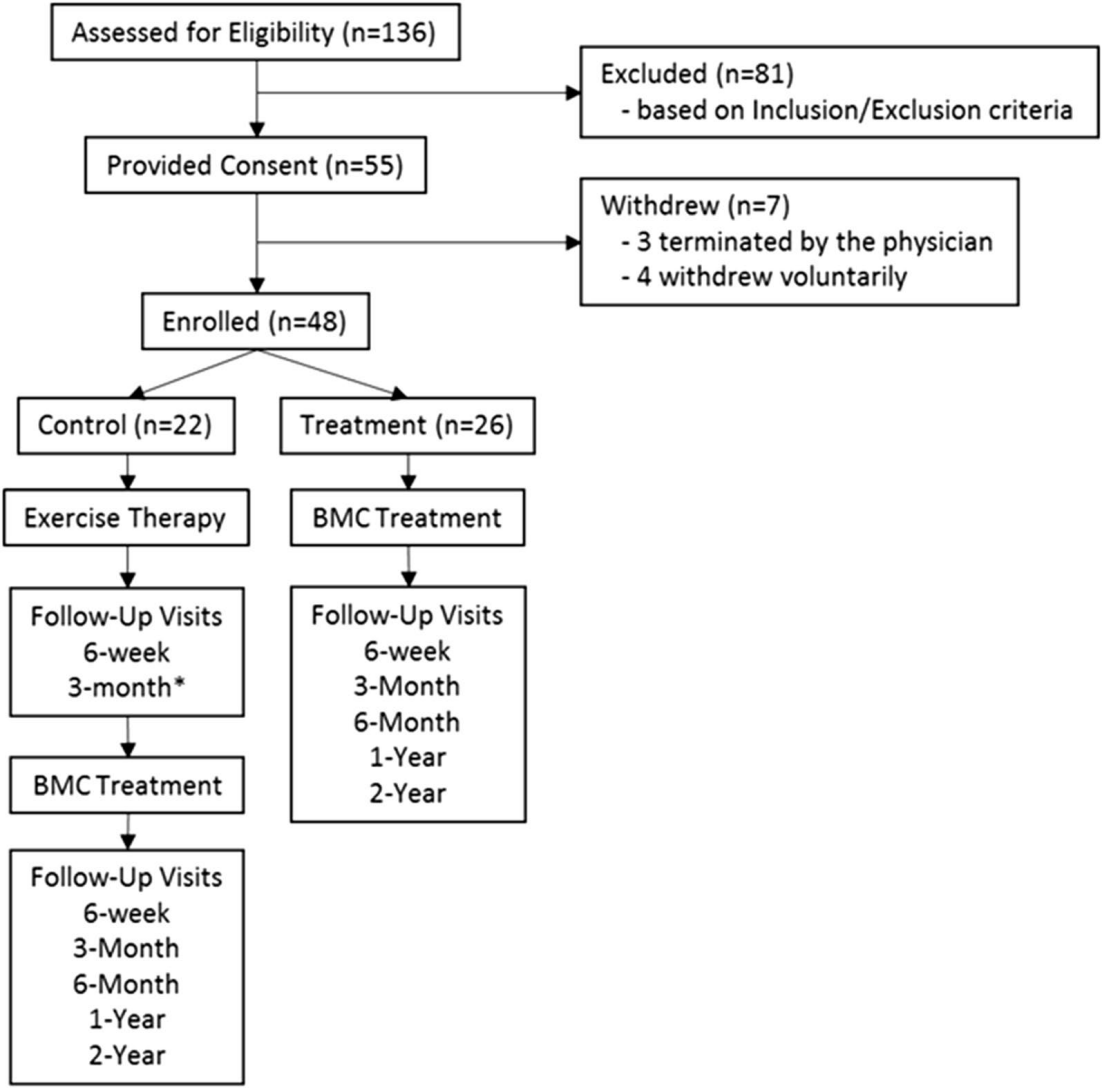
Study flow diagram

Of patients assessed for eligibility (n = 136), 55 met all inclusion criteria and provided consent. Four patients withdrew voluntarily after signing consent, but before receiving treatment (2 treatment; 2 exercise) and 3 were excluded for failing to comply with study requirements (2 treatment; 2 exercise). See Fig. 1 for a study flow diagram.

### BMC treatment procedure

Each patient in the treatment and crossover groups received a pre-treatment injection, bone marrow aspiration, BMC with platelet products injection, and a post-treatment injection.

#### Pre-treatment injection

The treatment process began 2–4 days prior to the BMC procedure with an injection of hyperosmolar dextrose. Using guided fluoroscopy, once Omnipaque contrast (NDC#0407-1411-20) was injected and confirmed as intra-articular, the solution consisting of 2–5 cc of 12.5% dextrose and 0.125% ropivacaine in normal saline was injected intra-articularly into the target sites of the knee with greatest cartilage loss.

#### Bone marrow aspiration, concentration and isolation of platelet products

A detailed description of the bone marrow aspiration (BMA) and concentration, platelet rich plasma and lysate preparation, and treatment injection procedures has been previously published [17]. Briefly, under imaging guidance, a total of 60–90 cc of BMA was drawn at a total of 6 aspiration sites on the posterior superior iliac crest. The BMA was processed by hand in a biologic safety cabinet to isolate the buffy coat to create BMC from which the total nucleated cell count was calculated. Concurrently, approximately 100 cc of venous blood was drawn and concentrated into two portions of leukocyte poor PRP by centrifuging the blood and extracting the plasma and buffy coat layers. One portion of PRP was set aside for injection and the other portion underwent further processing into platelet lysate (PL) via a freeze-thawing method [18].

#### BMC + platelet products injection

Using fluoroscopy, needle placement into the intra-articular space of the knee was confirmed by injecting a small amount of contrast (Omnipaque, NDC#0407-1411-20). A 5–7 cc injectate solution consisting of approximately 75% by volume of BMC, 12.5% by volume PRP, and 12.5% by volume PL was percutaneously injected, specifically targeting the sites of greatest chondral loss. Thereafter, patients were given a hinged unloader knee brace or a patella stabilizer brace (Fusion^®^ OA Plus or FreeRunner^®^, Breg, Inc., Carlsbad, CA, USA). The purpose of the brace was to off load the compartment treated to lengthen the reduced-load healing time.

#### Post-treatment injection

Two to four days after the BMC injection, the patient underwent an additional blood draw, from which approximately 3 cc solution of 25% by volume five times concentrated over baseline leukocyte poor PRP, 25% by volume of PL, 25% by volume of compounded 400 ng/ml dose of hydrocortisone, and 25% by volume of a 40 µg/ml dose of doxycycline, which was delivered via a percutaneous, ultrasound guided, intra-articular injection.

Patients followed a standard rehabilitation and return to activity protocol. Patients were instructed to wear a brace while weight bearing for 4 weeks and avoid any activities that caused more than 2/10 pain throughout rehabilitation. Days 0–3 patients were instructed to rest, restrict ambulation to household and community, and perform ROM exercises. From day 3 through week 6 therapeutic exercises included deep water emersion walking or jogging if patient had access to a pool for 30–45 min 3–5 times per week for 2 months. Stationary bike and then elliptical, as well as core training, non-resistance hip and knee strengthening were added as pain allowed. Weeks 6–12 patients could start walking for exercise, add resistance exercises/weight, hills, hiking, and low to moderate impact activity. Patients addressed weakness, ligament laxity and ROM deficits in PT. Week 12–26 patients were not given strict limitations and could gradually return to full activity as long as pain remains no more than 2/10.

### Exercise therapy

Due to patients’ geographical locations, physical therapy prescriptions were provided to patients with guidelines for the physical therapist to provide a home exercise program in an initial visit and an upgraded program at a 6-week follow-up visit. All programs followed the same basic principles of therapeutic exercise including functional strengthening, resistance training and monitor alignment for core, pelvis and entire lower extremity, as well as balance/neuro-muscular training, and aerobic activity based on what they had available (e.g. walk, stationary bike, water walk, etc.). If ROM was an issue, manual therapy and mobility was included.

### Outcome measures

Clinical outcomes were assessed before treatment and at each follow-up (Exercise: baseline and 3-months; Treatment: Baseline, 6-weeks, 3-, 6-, 12-, and 24-months). Adverse events were assessed at each follow-up visit via vital signs, physical examination, and self-reporting. Outcomes included the Knee Society Score of Assessment and Function (KSS), Visual Analogue Scale (VAS), Short Form-12 Scales (SF-12), and Lower Extremity Activity Scale (LEAS) [19–22]. The KSS consists of a KSS-knee and a KSS-function score, each on a 0 (poor) to 100 (excellent) scale, requiring input from both the patient and physician. A pain VAS is a self-reported subjective metric of pain intensity where “No pain” = 0 and “Pain as bad as it could possibly be” = 100, measured in millimeters. The SF-12 is comprised of physical (SF-12 Physical) and mental (SF-12 Mental) health summary scores, each ranging from 0 (low health)-100 (high health) points. The LEAS is a self-administered evaluation of activity on an 18-point scale ranging from 1 (confined to bed)-18 (vigorous physical activity). Patients were inquired about complications at every follow-up visit. Complaints reported outside of scheduled visits were followed-up in clinic when needed. Range of motion (ROM) in degrees, medication, age, gender, BMI, race, and bracing was also collected.

### Statistical analysis

Linear mixed-effects models with post hoc Tukey were used to determine outcome scores differences between time points for the treatment group. Three-month change scores (paired differences between post-treatment time points and baseline) were compared for control patients after they performed exercise, control patients after they received BMC treatment (crossovers) and treatment-only patients using an analysis of variance (ANOVA). If the ANOVA showed significant differences, post hoc t-tests were performed. Additionally, the treatment and crossover groups were combined and compared to the exercise group using 3-month change scores. The treatment and crossover groups were compared across all time points using linear mixed-effects models. Using similar models, outcomes between those with grade II and grade III OA were compared across all time points.

Exercise effectiveness was assessed via paired t-tests for all metrics between the 3-month time point and baseline. Differences with p < 0.05 were considered significant. All analyses were performed utilizing R, version 3.3.3, and RStudio, version 1.0.136.0.

## Results

### Demographics

Twenty-six patients were in the treatment group and 22 were in the control group, all whom crossed-over to the treatment group at 3-months. Four patients withdrew voluntarily; 2 at 3-months (treatment), 1 at 6-months after crossover, and 1 at 1-year (treatment). Seven patients were withdrawn by the investigator at the time point following additional treatments outside the study protocol (e.g. hyaluronic acid injections) (1 at 3-months; 3 at 6-months; 2 at 12-months; 1 at 24-months). Three patients received a total knee arthroplasty (TKA) and were withdrawn from the study at time of surgery (3-, 6- and 18-months). Baseline characteristics of gender, age, height, weight, BMI, KL grade and TNCC are shown in Table 2.

**Table 2:** Baseline demographic variables for treatment and exercise control groups

	Treatment			Control			*p*-value
	N	Average	SD	N	Average	SD	
Age (years)	26	54	8.9	22	57	8.5	0.17
BMI (lbs/in^2^)	26	26	2.9	22	26	2.9	0.84
Height (in.)	26	68	3.7	22	69	3.9	0.98
Weight (lbs.)	26	175	28	22	176	31	0.83
TNCC (million)	25	622	235	21	701	284	0.42

	N	N	%	N	N	%	
KL OA grade	26			22			
Grade II		11	42		10	45	
Grade III		15	58		12	55	

*TNCC* total nucleated cell count, *KL* Kellgren–Lawrence OA grading scale

### Adverse events

No serious adverse events were identified in any study patients during follow-up for either group. The most common complaint was pain after treatment (16 patients), while one patient reported swelling and grinding with pain, and another had a persistent popliteal fossa fluid accumulation, which was aspirated. Patients reporting recurrent knee pain after the BMC treatment were given PRP injections at the discretion of the treating physician (15 = 1 PRP; 2 = 2 PRP treatments) at the following time points: 3-month (N = 4); 6-months (N = 3); 12-months (N = 10); 18-months (N = 1); 24-months (N = 1).

### Clinical outcomes

Comparing the exercise therapy group (N = 22) to the BMC treatment (N = 24) at 3-months, patients who received BMC showed significant improvement in LEAS (p < 0.01) and KSS-knee scores (p < 0.001) over those who followed a home exercise therapy program. There were no significant differences between groups on VAS pain, KSS-function, SF-12, or ROM. See Table 3 for the changes on outcome measures from baseline to 3-months.

**Table 3:** Exercise therapy versus BMC treatment 3-month changes on outcome measures

Metric	Group	N	3-month change score	*p*-value
VAS (mm)	Exercise	22	− 8	0.40
	BMC	24	− 12.5	
LEAS	Exercise	21	− 1.1	0.002
	BMC	24	0.8	
KSS—knee score	Exercise	22	0.6	< 0.001
	BMC	23	12.0	
KSS—function score	Exercise	22	2.3	0.17
	BMC	24	7.5	
SF-12 physical	Exercise	22	2.4	0.27
	BMC	24	4.9	
SF-12 mental	Exercise	22	− 1.5	0.68
	BMC	24	− 2.4	
ROM (degrees)	Exercise	22	2.6	0.97
	BMC	23	2.6	

*p*-value between groups

**Fig. 2:**
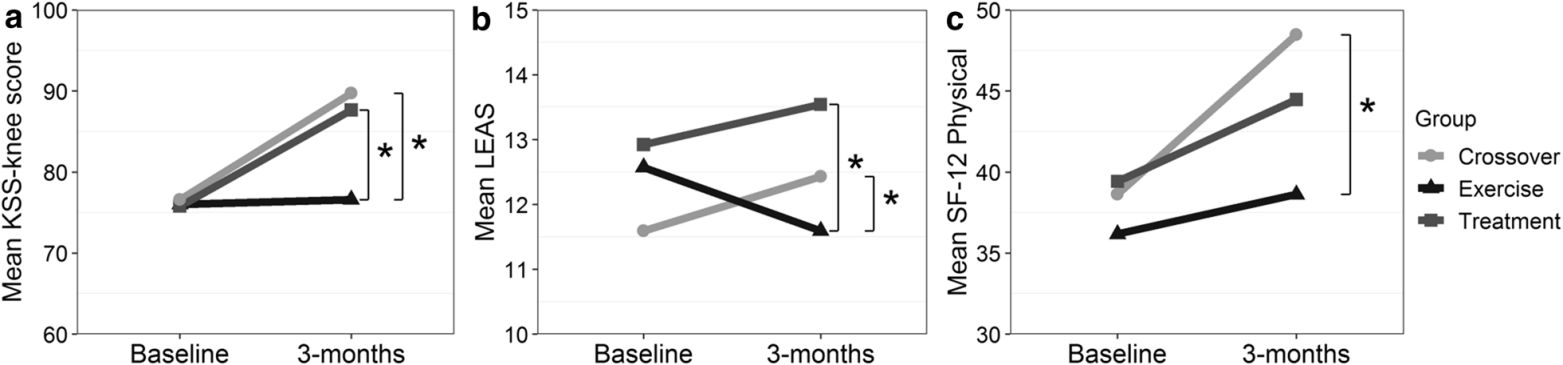
Average scores at baseline and 3-months for exercise, crossover, and treatment groups. *p < .05 for **a** KSS-knee, **b** LEAS, **c** SF-12

To determine if patients who crossed-over into the treatment group after undergoing exercise therapy differed from those receiving exercise alone or BMC alone, these three groups were compared separately. Three-month change scores differed significantly between the treatment group, exercise group, and crossover group for KSS-knee scores, SF-12 Physical, and LEAS (Fig. 2). The crossover group’s KSS-knee score (p = 0.002), SF-12 Physical (p = 0.018), and LEAS (p = 0.004) change scores all improved significantly after patients received treatment compared to when they had only performed exercise. Change scores at 3-months for KSS-knee score (p < 0.001) and LEAS (p = 0.002) were significantly better for the treatment group compared to the exercise group. No significant differences were seen for 3-month change scores between the treatment group and the crossover group for KSS-knee score, SF-12 Physical, and LEAS (p > 0.05).

Models showed no significant differences in any outcome metrics over time between those who received treatment only versus treatment after exercise (crossovers). The effect of exercise was assessed by comparing exercise group scores after 3-months of exercise to their baseline. The 3 months of exercise therapy resulted in significant improvements in LEAS (p = 0.015) and ROM (p = 0.022). No significant differences were found between patients with Grade 2 versus Grade 3 OA for any metric (p > 0.05).

**Fig. 3:**
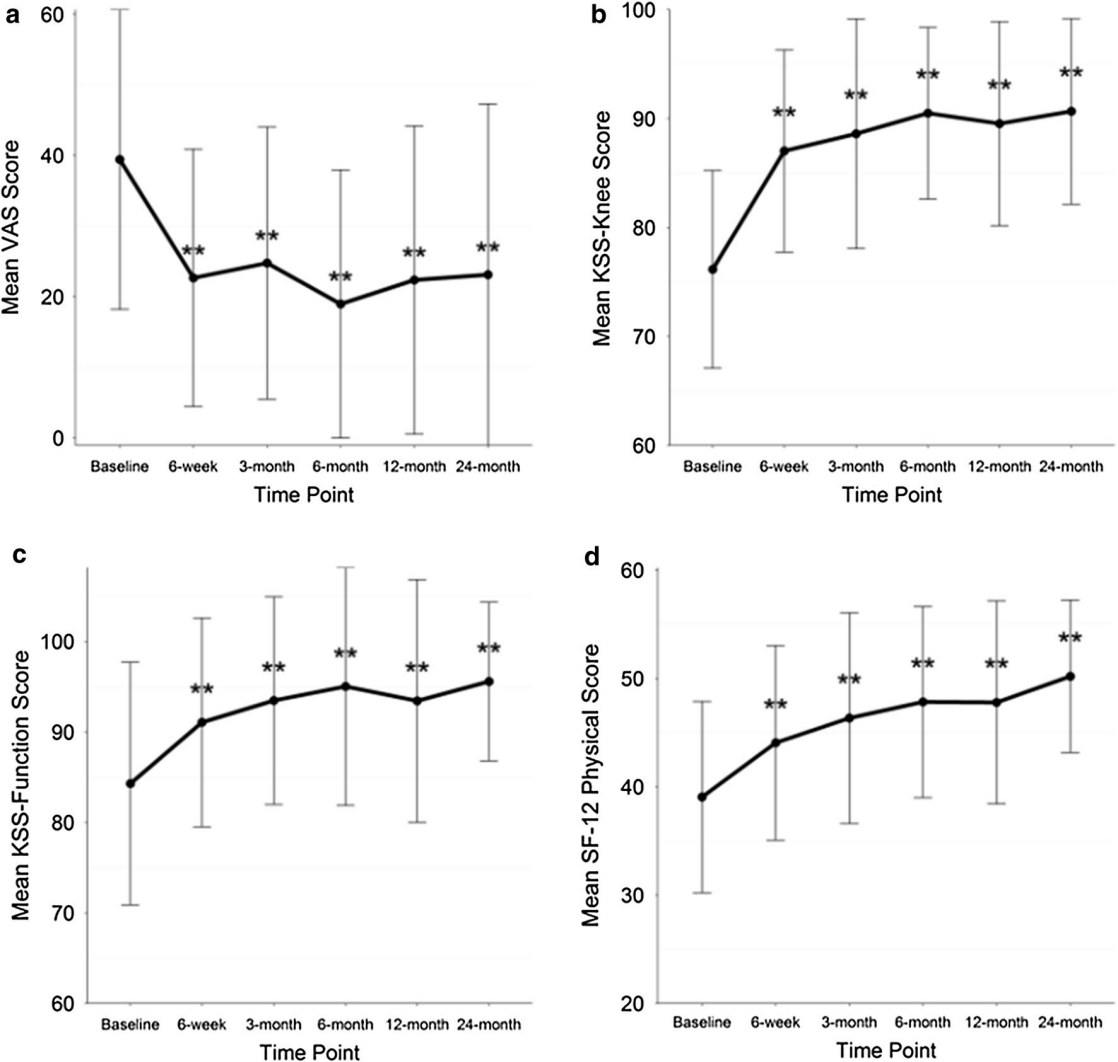
Average clinical outcome scores with standard deviation bars. Significant differences from baseline **p < .01, for **a** VAS; **b** KSS-knee score; **c** KSS-function score; and **d** SF-12 Physical

After BMC treatment (combining BMC and crossover groups), all metrics improved significantly across time [KSS-knee score, KSS-function score, SF-Physical, LEAS, and ROM (p < 0.001)]; except for SF-12 Mental scores (p = 0.071) (Fig. 3). Post-hoc Tukey showed VAS was significantly lower (p < .0001), while KSS-knee score, KSS-function score and SF-12 Physical averages were significantly higher (p < 0.001) at all post-treatment time points compared to baseline. LEAS averages were significantly higher at 6, 12 and 24 months (p < 0.01) versus baseline. ROM averages were significantly higher (p < 0.05) at 3, 6 and 12 months than before treatment. Change scores for all metrics can be seen in Table 4.

**Table 4:** Clinical outcome baseline and change score averages at each post-treatment visit (treatment and crossover data combined)

Metric	Visit	N	Mean/change score*	SD	*p*-value	Metric	Visit	N	Mean/change score*	SD	*p*-value
VAS (mm)	Baseline	48	39	21		LEAS	Baseline	48	12	3	
	6-week	48	− 17	18	< 0.001		6-week	48	0.3	2	0.903
	3-month	46	− 14	16	< 0.001		3-month	46	0.8	2	0.081
	6-month	45	− 19	17	< 0.001		6-month	45	1.1	3	0.007
	1-year	43	− 15	20	< 0.001		1-year	43	1.1	2	0.004
	2-year	42	− 14	24	< 0.001		2-year	42	1.2	3	0.004
KSS-knee score	Baseline	48	76	9		KSS-function score	Baseline	48	84	13	
	6-week	47	11	9	< 0.001		6-week	48	7	13	< 0.001
	3-month	44	13	11	< 0.001		3-month	46	9	12	< 0.001
	6-month	45	14	8	< 0.001		6-month	45	9	13	< 0.001
	1-year	43	11	12	< 0.001		1-year	43	7	13	< 0.001
	2-year	42	13	11	< 0.001		2-year	42	8	12	< 0.001
SF-12 physical	Baseline	48	39	9		SF-12 mental	Baseline	48	58	6	
	6-week	48	5	9	< 0.001		6-week	48	1.0	7	0.918
	3-month	46	7	9	< 0.001		3-month	46	1.4	6	0.714
	6-month	45	8	11	< 0.001		6-month	45	1.8	9	0.340
	1-year	43	7	10	< 0.001		1-year	43	2.2	9	0.138
	2-year	41	9	11	< 0.001		2-year	41	2.6	10	0.055
ROM (degrees)	Baseline	48	131	9							
	6-week	47	1.5	6	0.713						
	3-month	44	3.0	6	0.052						
	6-month	45	4.0	6	0.001						
	1-year	43	2.5	9	0.205						
	2-year	42	1.2	8	0.895						

*p*-value compared to baseline scores

* Values for baseline are means and for all follow-up time points are change score means

## Discussion

Patients who received a specific protocol of BMC and platelet products improved significantly in activity levels (shown by LEAS), as well as pain, ROM and stability as assessed by the KSS-knee score compared to patients who underwent a home exercise therapy program for 3 months for the treatment of moderate knee OA. Pain decreased for both the exercise therapy and the BMC groups, and function increased for the BMC group (KSS-function), although did not differ significantly between the 2 groups. Exercise therapy provided significant improvements in ROM and activity levels at 3-months compared to baseline, albeit a lower activity level than the BMC treatment produced.

All individuals in the exercise therapy groups chose to crossover and receive BMC treatment. Since this crossover group did not differ significant from the BMC only group, data was combined to determine long-term efficacy throughout the 2-year study duration. Significant improvements in pain and functionality were maintained through 2 years follow-up after receiving BMC treatment. These findings are in-line with several previous studies that suggest BMC as an alternative treatment for knee osteoarthritis [17, 23].

To our knowledge, this is the first prospective randomized controlled trial comparing the use of autologous BMC and exercise therapy for knee OA. A primary objective for this investigation was to determine how a cross-section of patients who present with knee OA, including patients with varying degrees of pain and functional levels, respond to a specific protocol of BMC and platelet product treatment. The most frequently reported side effect of treatment was temporary pain and swelling, which may be explained by the release of trophic factors associated with the intra-articular injection of MSCs, a phenomenon observed in animal models [24]. It is important to highlight that even though 52% of patients were classified as K-L grade 3 and deemed total knee arthroplasty candidates, only 3 patients received TKA during the study follow-up period, and no other surgeries were reported.

Only one other randomized trial investigating the use of BMC for knee OA has been published. Shapiro et al. described a trial of BMC in one knee compared to saline placebo in the contralateral knee in bilateral knee OA patients and concluded that BMC was no more effective than saline [14]. Several factors may account for the differences in the present study versus the Shapiro et al. study. It appears that the cell counts were substantially (~ 75%) lower than in the present investigation, and likely fell below a previously published threshold needed to produce significant symptom improvement [25]. Additionally, Shapiro and colleagues’ injectate consisted of 33% BMC, which was less than half the proportion used in the present investigation (which was 75%). The remaining volume of injectate used by Shapiro et al. consisted of platelet poor plasma (PPP), compared to the PRP and PL in present study, which contain greater quantities of platelet-derived growth factors compared to PPP [26].

The exact properties responsible for the beneficial effects of treatment with BMC for knee OA are currently unknown. The pathogenesis of OA is associated with an alteration in the repair and breakdown of the articular cartilage, which is believed to be influenced by multiple factors including the depletion of healthy MSCs in the microenvironment [27]. BMC contains MSCs and other regenerative factors including hematopoietic stem cells (i.e. pluripotent cells), platelets containing over 1500 growth factors, white blood cells, macrophages, and several different cytokines (e.g. interleukin 1-receptor antagonist protein and alpha-2-macroglobulin) [12]. Hence, the anti-inflammatory and immunomodulatory effects of BMC may lead to the regeneration of damaged tissue, modification of the microenvironment to aid in cartilage regeneration, and/or simply pain and inflammatory modulation. Paracrine factors may also play a role in potential therapeutic mechanisms [28].

In the present investigation, we used a pre- and post-injection protocol to prime the knee before receiving the BMC as well to aid in proliferation of MSCs after receiving the BMC. The rationale for an inflammatory pre-injection was to stimulate local MSCs. For example, MSCs have been found in higher concentrations in the synovial fluid in an acutely injured knee compared to a normal knee [29]. Doxycycline was added to the injectate because as it has been shown to decrease catabolic cytokines (matrix metalloproteinases) and improve MSC induced chondrogenesis [30]. Nanogram dosing of corticosteroids has been previously shown to promote chondrogenesis with limited systemic response [31]. The addition of autologous PRP and PL for the post-injection is primarily to aid in the proliferation of both native MSCs and those contained in the BMC. The benefits of PL as a culture medium for MSCs and its effects on cellular proliferation have also been widely reported [32].

There are several potential limitations of the present study to consider. First, there were 17 patients that received PRP injections after undergoing the BMC treatment protocol for recurrent pain. PRP used alone has been previously shown to have a limited effect on moderate OA [33]. Second, doxycycline and ultra-low dose corticosteroid used in post-injection protocol theoretically may have contributed to the observed effects via their mechanisms of decreasing matrix metalloproteinases and aid in local chondrocyte proliferation, respectively, although is unlikely to account for the observed results [30, 34]. A third limitation is the relatively short duration of the exercise therapy group and the allowance of those in the exercise group to crossover and receive BMC treatment. This was designed to aid in study participant recruitment and retention. Statistically, the crossover group did not improve more than the BMC only treatment group. A final limitation is missing data at some follow-up time points due to being lost to follow-up as well as patients that were removed for receiving treatments outside of the study protocol. Approximately 20% of patients received outside treatment (hyaluronic acid injections or TKA) during the study, suggesting that a portion of the study sample did not achieve the desired clinical response after the BMC and platelet product treatment. Since we believe that these outside treatments would affect clinical outcomes, we did not include data at subsequent follow-up time points for a patient after the time of the outside intervention. Further research focused on identifying good or poor candidates for this treatment is needed. Future investigations should also include patients that completed physical therapy for 6–12 months.

## Conclusion

To our knowledge, the present study is the first randomized trial comparing a specific protocol of BMC with platelet products to exercise therapy for the treatment of knee osteoarthritis. While exercise therapy helped knee OA symptoms and function, this specific protocol of intra-articular injection of BMC with platelet products had a greater impact on patients. The results of our study warrant expanded investigation.

## Abbreviations

ACL: anterior cruciate ligament
ANOVA: analysis of variance
BMC: bone marrow concentrate
BMI: body mass index
IRB: Institutional Review Board
KL: Kellgren–Lawrence
KSS: Knee Society Score
LEAS: Lower Extremity Activity Scale
MSC: mesenchymal stem cells
NSAID: non-steroidal anti-inflammatory drugs
OA: osteoarthritis
PL: platelet lysate
PPP: platelet poor plasma
PRP: platelet-rich plasma
ROM: range of motion
SF-12: Short form-12 Scales
TKA: total knee arthroplasty
TNCC: total nucleated cell count
VAS: Visual Analogue Scale
